# Models for Natural Killer Cell Repertoire Formation

**DOI:** 10.1080/10446670310001642140

**Published:** 2003

**Authors:** Mali Salmon-Divon, Petter Höglund, Ramit Mehr

**Affiliations:** 1 Faculty of Life Sciences Bar-Ilan University Ramat-Gan 52900 Israel; 2 Microbiology and Tumor Biology Center Karolinska Institutet Stockholm S-171 77 Sweden

## Abstract

Natural killer (NK) cells lyse only cells that do not express sufficient levels of self class I MHC molecules. Inhibition of lysis is mediated by inhibitory receptors expressed by NK cells, such as the murine Ly49 receptors, that bind to MHC class I molecules. Since inhibitory receptor genes and MHC class I genes are located on different chromosomes, and are hence not automatically co-inherited, NK cells apparently adapt to the MHC environment during their development. Two models have been proposed to account for this “education” process of NK cells. The *two-step selection* model postulates that developing NK cells initiate the stable expression of a random set of Ly49 genes, and then undergo two selection steps, one for cells that express a sufficient number of self-MHC receptors, and one against cells that express too many inhibitory receptors. The *sequential* model postulates that a cell keeps initiating the stable expression of additional inhibitory receptors until a sufficient expression level of self-MHC specific receptors is reached, and the cell matures. In this study we implement both models in computer simulations, and compare simulation results to experimental data, in order to evaluate the relative plausibility of the two models.


*Clinical & Developmental Immunology*, June–December 2003 Vol. 10 (2–4), pp. 183–192

Taylor&Francis healthsciences

### Models for Natural Killer Cell Repertoire Formation

MALI SALMON-DIVON[a], PETTER HÖGLUND[b] and RAMIT MEHR[a,*]

[a]*Faculty of Life Sciences, Bar-Ilan University, Ramat-Gan 52900, Israel;* [b]*Microbiology and Tumor Biology Center, Karolinska Institutet, S-171 77, Stockholm, Sweden*

Natural killer (NK) cells lyse only cells that do not express sufficient levels of self class I MHC molecules. Inhibition of lysis is mediated by inhibitory receptors expressed by NK cells, such as the murine Ly49 receptors, that bind to MHC class I molecules. Since inhibitory receptor genes and MHC class I genes are located on different chromosomes, and are hence not automatically co-inherited, NK cells apparently adapt to the MHC environment during their development. Two models have been proposed to account for this "education" process of NK cells. The *two-step selection* model postulates that developing NK cells initiate the stable expression of a random set of Ly49 genes, and then undergo two selection steps, one for cells that express a sufficient number of self-MHC receptors, and one against cells that express too many inhibitory receptors. The *sequential* model postulates that a cell keeps initiating the stable expression of additional inhibitory receptors until a sufficient expression level of self-MHC specific receptors is reached, and the cell matures. In this study we implement both models in computer simulations, and compare simulation results to experimental data, in order to evaluate the relative plausibility of the two models.

*Keywords*: Computer simulations; Ly49 receptors; Mathematical models; Natural killer cells

### INTRODUCTION

Natural killer (NK) cells are large granular lymphocytes which can lyse tumor and virus-infected cells, and can mediate acute rejection of bone marrow cell grafts. NK cells are able to efficiently lyse targets that lack expression of MHC antigens but usually do not lyse target cells expressing sufficient levels of self-MHC. This is explained by the "missing self hypothesis" (Ljunggren and Kärre, 1990), which proposes that the ability of NK cells to destroy certain cells appears to be regulated by a balance between activating and inhibitory signals transduced by activating and inhibitory receptors. Support for the "missing self hypothesis" has been provided by the identification and cloning of membrane receptors on NK cells (Ly49 receptors in rodents and KIRs receptor in primates), that bind MHC class I molecules on potential target cells and inhibit NK cell-mediated cytotoxicity (Colonna and Samaridis, 1995). In this study we focus on the development of the Ly49 receptor repertoire.

Different Ly49 genes are expressed in overlapping subsets within the total NK cell population, providing for a diverse repertoire of Ly49 receptors (Kubota *et al.*, 1999). Among Ly49 genes, Ly49-a,b,c,e,f,g,i,j,o,q,s,t and v are predicted to code for inhibitory receptors, while Ly49-d,h,k,l,m,n,p,r,u and w are predicted to code for activators (Anderson *et al.*, 2001). Inhibitory Ly49s contain immune receptor tyrosine based inhibitory motif (ITIM) in their cytoplasmic region (Smith *et al.*, 1994; Anderson *et al.*, 2001), providing a mechanism by which inhibitory Ly49s might inhibit NK cell activation. Activating Ly49s lack an ITIM and instead associate with the signal-transducing protein DAP12 (Mason *et al.*, 1996) which contains an immunoreceptor tyrosine-based activation motif (ITAM) and transmits activating signals.

NK cell receptors recognize specific alleles of class I heavy chains that form a tri-molecular complex with $\beta_2 m$ and peptide. The known specificities of the Ly49 receptors are listed in Table I. Earlier reports have shown that the repertoire of inhibitory receptor expression by NK cells is influenced by MHC class I expression in the host (Höglund *et al.*, 1988; Öhlén *et al.*, 1989; Held *et al.*, 1996; Held and Raulet, 1997; Salcedo *et al.*, 1997). Since Ly49 genes and class I genes are located on different chromosomes (Yokoyama *et al.*, 1990), it appears that Ly49 receptors are not automatically co-inherited with cognate class I genes. Based on this observation, it is believed that NK cells adapt to the MHC environment during their development or maturation.

Two possibly interrelated processes have been proposed as explanations of how the receptor repertoire of NK cells is generated and shaped: (a) the molecular mechanisms that activate transcription of different receptor genes in different cells; (b) an "education" system that shapes

*Corresponding author. Tel.: +972-3-531-7990. Fax: +972-3-535-1824. E-mail: mehrra@mail.biu.ac.il

ISSN 1740-2522 print/ISSN 1740-2530 online © 2003 Taylor & Francis Ltd
DOI: 10.1080/10446670310001642140

TABLE I The known MHC specificities of Ly49 receptors

| Activating receptors | | Inhibitory receptors | |
|---|---|---|---|
| Receptor | MHC specificity | Receptor | MHC specificity |
| Ly49D | $D^d, D^r, Dsp2$ | Ly49A | $D^d, D^k, D^b$ and MHC Ags of the H-$2^{f,q,r,s,v}$ |
| Ly49H | $D^b$ | Ly49B | ? |
| Ly49K* | ? | Ly49C | $K^d, D^d, D^k$ and MHC Ags of the H-$2^{b,f,q,r,s,v}$ |
| Ly49L | H-$2^K$ | Ly49E | ? |
| Ly49N* | ? | Ly49F | weakly to H-$2^d$ |
| Ly49M | ? | Ly49G | $D^d, L^d$ |
| Ly49P | $D^d$ | Ly49I | $K^b, K^d$ and MHC Ags of the H-$2^{b,d,q,r,s,v}$ |
| Ly49R | $D^d, D^k, L^d$ | Ly49J | ? |
| Ly49U | ? | Ly49O | $D^b, D^d, D^k, L^d$ |
| Ly49W | H-$2^d$, H-$2^k$ | Ly49Q | ? |
| | | Ly49S | ? |
| | | Ly49T | ? |
| | | Ly49V | H-$2^b$, H-$2^d$, H-$2^k$ |

* No complete protein coding sequence is known.

the repertoire based on the MHC class I molecules expressed by the host. This system ensures that each functional NK cell expresses at least one self-class I-specific inhibitory Ly49 receptor (to prevent auto-aggression), but not too many self-class I-specific receptors, which would have made such cells useless by preventing them from attacking self-cells that had extinguished expression of only one class-I molecule. In this study we focus on NK cell education, assuming the pre-selection probabilities of expression of each receptor are known.

Two schemes were proposed to describe how NK cell education might proceed. The first is the *Sequential* activation model. In this scheme, Ly49 genes are stably activated (that is, once a gene is activated, it stays "on") in developing NK cells continuously and cumulatively, but in random order. The cells are periodically tested for interaction with self-class I molecules on neighboring cells. Strong interaction between a single type of Ly49 receptor and self-molecules prevents new receptors from being expressed and results in maturation of the cell. Weak interaction may not be sufficient to prevent new receptor gene expression. In this scheme, a single testing step accomplishes both tasks of the education process; however, this single step may be repeated many times during the cell's development.

The second scheme is the *Two-step selection* model for NK cell repertoire formation. In this model, the repertoire is fully formed at an initial stage by a stochastic process and subsequently shaped by two selection steps: one selects for cells expressing at least one self-specific receptor, and the other selects against cells expressing too many self-specific receptors. The two-step selection thus occurs only once for each cell, when it has completed its receptor gene activation. Depending on the signaling thresholds of these steps, the process may allow maturation of cells expressing more than one self-specific receptor. In many cases, the frequencies of cells expressing more than one receptor can be approximated by the product of the expression frequencies of the individual receptors (Kubota *et al.*, 1999). However, deviations from this "product rule" have been observed (Smith *et al.*, 2000), which point at either the effects of selection, or sharing of expression mechanisms between genes, or both.

Mathematical modeling and computer simulations are used in this study to evaluate the hypothesized selection mechanisms that shape the natural killer cell repertoire. A similar approach was previously used by Vance and Raulet (1998) to evaluate the two education models, based on several simplified assumptions. One was that the probability of being expressed is initially the same for all receptors. They thus ignored the possible consequences of the molecular process that controls receptor gene transcription initiation, occurring before education is expected to occur. In addition, Vance and Raulet assumed in their model that a given receptor, in a binary fashion, either binds or does not bind to self-MHC class I molecules, regardless of the effect of the binding affinity. However, binding affinity may be critical for determining the signaling thresholds of the selection process.

Recently, it has been shown that an NK cell may express between three to seven class I-specific receptors—inhibitory as well as activatory (Kubota *et al.*, 1999), in a mono-allelic manner (Held *et al.*, 1999). However, little is known about how combinatorial expression of different class-I specific receptors by an individual NK cell contributes to its function and self-tolerance. Lately, it has been demonstrated (Hanke and Raulet, 2001) that $D^d$ co-recognition by Ly49A and Ly49G2 inhibits NK cell cytotoxicity more strongly than recognition by either Ly49A or Ly49G2 alone. This finding prompted (Hanke and Raulet, 2001) to raise the possibility that inhibition of NK cells by self cells is not necessarily attributable to one single receptor–ligand interaction, but is rather a result of cumulative signaling through several receptors. Thus, weak interaction between the Ly49 receptor and self-MHC class I molecules might be insufficient to prevent new receptor gene expression in the sequential model, or might allow maturation of NK cells that express more than one self-specific Ly49 receptor in the two-step selection model. Therefore, when modeling quantitatively the effect of the selection model on the final NK cell repertoire, it is necessary to consider the cumulative effects of several interactions accounting for all the self receptors expressed by each NK cell.

The study by Vance and Raulet (1998) was over-simplified and hence did not generate any testable predictions. In a previous study (Salmon-Divon *et al.*, 2002), we used a more extended mathematical formulation of the pre- and post-selection receptor expression patterns in the NK cell repertoire, for the general case in which the pre-selection frequencies are not necessarily the same for all receptors. The two-step selection model was implemented as a computer program which calculated the post-selection frequencies based on the mathematical formulation. The sequential model was implemented as a computer simulation of a population of cells developing in time and being continually selected. We have addressed the question: do the two different models for NK cell education give different dynamics of NK cell development, or different repertoire compositions? Our results have shown that the simple versions of the two models which we have used, in which all inhibitory receptors were treated in a binary fashion as either binding or not binding to each self MHC class I molecule, are insufficient to distinguish between the two models.In the present study we extend our previous models to the case in which the binding affinities of Ly49 receptors to their ligands are not necessarily equal. We examine the effects of variations in receptor binding affinity on the previous predictions, and ask whether, under these conditions, the predictions of the two models differ. As will be shown below, the refined models generate testable predictions that will enable the experimentalists to differentiate between the two education models.

### RESULTS

### Using Data from MHC Class I-deficient Mice to Approximate Pre-selection Frequencies

The NK cell repertoire in MHC class I-deficient mice should provide information on the pre-selection NK cell repertoire. One might expect MHC class I-deficient mice to be incapable of recognizing "missing self": the sequential model predicts that all NK cells in such mice should express all Ly49 receptors, and the two-step selection model would predict that such mice have no NK cells. The phenotype of NK cells in $\beta_2m^-$ and $TAP^-$ mice is incompatible with either model. These mice contain normal numbers of cells with the NK phenotype (Höglund *et al.*, 1991; Liado *et al.*, 1991), but these cells are defective in recognition of class I-deficient and class I-allogeneic cells (Bix *et al.*, 1991; Höglund *et al.*, 1998). The frequencies of NK cells expressing each of several Ly49 receptors in these mice were marginally higher, and the frequencies of NK cells co-expressing various receptor pairs or trios were substantially higher than in MHC class $I^+$ mice (Held *et al.*, 1996; Salcedo *et al.*, 1997). It is known that $\beta_2m^-$ and $TAP^-$ mice are not completely class I-deficient, as they express on their cell surface low levels of functionally conformed class I molecules. In spite of that, one can take the frequencies of cells expressing each of the Ly49 receptors in those mice as a first approximation for the values of activation probabilities of each receptor gene. Knowledge of the activation probabilities allows us to predict the post-selection repertoire in non-MHC-deficient mice according to either selection model, using the computer simulations described in the Methods section.

### Prediction of NK Cell Repertoire Compositions: A Sample Case

Comprehension of the parameters governing NK cell repertoire, such as receptor binding affinity to MHC molecules, is essential for understanding the processes that shape the NK cell repertoire in general, and can help us evaluate the relative plausibility of each education model. In order to demonstrate the results generated by our simulations, we show here the results of one run of each model, based on a sample set of parameters (Table II). Table III contains the repertoires that had been generated according to the two models under the parameters given in Table II, with the minimum value of receptor-MHC binding affinity required for NK cell maturation, $A_{\min} = 4$, for both the sequential and the two-step selection model, and with $A_{\max} = 5$ or 6 in the two-step selection model. Since in our particular set of parameters (Table II) the affinity levels of the four receptors are 0,1,2 and 3, and $A_{\min} = 4$, none of the receptors itself is sufficient to bring about the maturation of a cell. All expression patterns that have a total affinity under four hence have a frequency $= 0$ in the post-selection repertoire under both models. In the example with $A_{\max} = 6$, since this value allows expression of all the receptors together, the same subsets of receptors that are allowed in the sequential model also appear in the two-step selection model, albeit with different frequencies. Although receptor C is assumed to be non-self, and hence does not affect the selection process in the two-step selection model, the final frequencies of cases that differ only in whether C is expressed or not differ (compare, e.g. line 10–14), due to differences in their pre-selection frequencies. In the example with $A_{\max} = 5$, cases with total affinity of six are allowed in the sequential model but not in the two-step selection model; hence, here we see a larger difference between the two models.

TABLE II A sample set of parameters used in a simulation*

| Gene | Specificity | Pre-selection expression probability | Affinity |
|---|---|---|---|
| A | Self | 0.7 | 1 |
| B | Self | 0.8 | 2 |
| C | Non self | 0.6 | 0 |
| D | Self | 0.3 | 3 |

* In this sample set there were four genes, three of which encode for self-MHC-receptors and one for a non-self receptor.

TABLE III The post-selection repertoires that had been shaped according to each model

| Expression pattern | | | | Frequency | | |
|---|---|---|---|---|---|---|
| | | | | | Selection model | |
| A | B | C | D | Sequential model $A_{min} = 4$ | $A_{min} = 4$ $A_{max} = 6$ | $A_{min} = 4$ $A_{max} = 5$ |
| $X$ | $X$ | $X$ | $X$ | 0 | 0 | 0 |
| √ | $X$ | $X$ | $X$ | 0 | 0 | 0 |
| $X$ | √ | $X$ | $X$ | 0 | 0 | 0 |
| √ | √ | $X$ | $X$ | 0 | 0 | 0 |
| $X$ | $X$ | √ | $X$ | 0 | 0 | 0 |
| √ | $X$ | √ | $X$ | 0 | 0 | 0 |
| $X$ | √ | √ | $X$ | 0 | 0 | 0 |
| √ | √ | √ | $X$ | 0 | 0 | 0 |
| $X$ | $X$ | $X$ | √ | 0 | 0 | 0 |
| √ | $X$ | $X$ | √ | 0.09366 | 0.05957 | 0.147368 |
| $X$ | √ | $X$ | √ | 0.11072 | 0.10212 | 0.252632 |
| √ | √ | $X$ | √ | 0.09437 | 0.23829 | 0 |
| $X$ | $X$ | √ | √ | 0 | 0 | 0 |
| √ | $X$ | √ | √ | 0.09884 | 0.08936 | 0.221053 |
| $X$ | √ | √ | √ | 0.11329 | 0.15319 | 0.378947 |
| √ | √ | √ | √ | 0.49902 | 0.35744 | 0 |

The post-selection repertoires were derived under the parameter values defined in Table II. √ represents an expressed receptor while $X$ represents a non-expressed receptor, so that we show the predicted frequencies of all possible combinations (expression patterns) of the four receptors. In the three simulations shown, the value of $A_{min}$ was 4 for both the sequential and the two-step selection model. In one simulation of the two-step selection model the value of $A_{max}$ was 6, and in the second, $A_{max} = 5$.

### NK Cell Repertoire Compositions Generated by the Two Education Models

The actual post-selection repertoire could be predicted, using our simulations, only if the values of gene activation probabilities and receptor binding affinities were known. However, sufficient information on these parameters is still unavailable in most cases; only the NK receptor distribution in the final repertoire is available. Hence, we searched in our simulations for sets of parameters, if there are any, which lead to a final repertoire that is close to that found in laboratory experiments, such as the data by Hanke *et al.* (2001). To find the correct set of parameters for each case, we repeated our simulations for all possible cases of $A_{min}$, $A_{max}$, all possible values of affinity levels for all receptors, and all possible expression patterns of the four receptors. Among the results of all these runs, we searched for the sets of parameters whose final repertoire gives the best fit to that found in laboratory experiments, as defined in the Methods section.

For the sake of comparison of our results to experimental data, we had to decide whether the expression frequencies of each receptor (or receptor pair) in (Hanke *et al.*, 2001) represent, and should be compared with, the overall frequencies of expression of the corresponding receptor (or pair) regardless of what other receptors the cells may express (the "inclusive" case), or with the frequencies of cells expressing only the indicated receptor or pair (the "exclusive" case). The two cases obviously have different frequencies for each gene or gene pair considered. Since the experiments in (Hanke *et al.*, 2001) used a four-color flow cytometric analysis, with two colors used to identify NK1.1$^{+}$CD3/8$^{-}$ cells, and the remaining two to identify the receptors, the experimental data should apparently be taken to represent inclusive expression frequencies. However, as there is no information on co-expression of more than two receptors, the data do not indicate whether these cases are rare (in which case the exclusive case will be more realistic) or not (in which case the inclusive case is more likely to reflect the correct frequencies). Simulations in the inclusive mode gave the best fits to experimental data (Fig. 1).

One observation that can be made here is that the best fit and highest score, in both models, were usually obtained with $1 \leq A_{min} \leq 4$; this is because low $A_{min}$ allows more cells to mature, such that a case that fits the experimental results is more likely to be found. The best fit and highest score for each $A_{min}$ value were very well correlated. Since we found that either one of the two NK cell education models can account for the experimental receptor frequencies in the final repertoire under specific sets of parameters, we conclude that one cannot use these data to support or refute either model.

### Predictions of Receptor-Ligand Reactivities Generated by Our Simulations

How, then, can we decide between the two education models? The decision between them may come from examination of the parameter values, such as receptor binding affinities, which lead to the best fit in each model, and comparison of these parameters to experimental observations. That is, we may examine the values of receptor binding affinities of Ly49 receptors to H-2 molecules in the case with the best fit to experimental data, generated by each education model.

For instance, it is known that H-2$^{k}$ molecules serve as a functional ligand for Ly49A and Ly49I; however, no H-2$^{k}$ reactivity has been reported for Ly49G2 and Ly49F. Although we ran the programs for all possible cases and the whole range of affinity values, good fits to experimental results were, indeed, only obtained when receptors A and I were defined as self receptors in the H-2$^{k}$

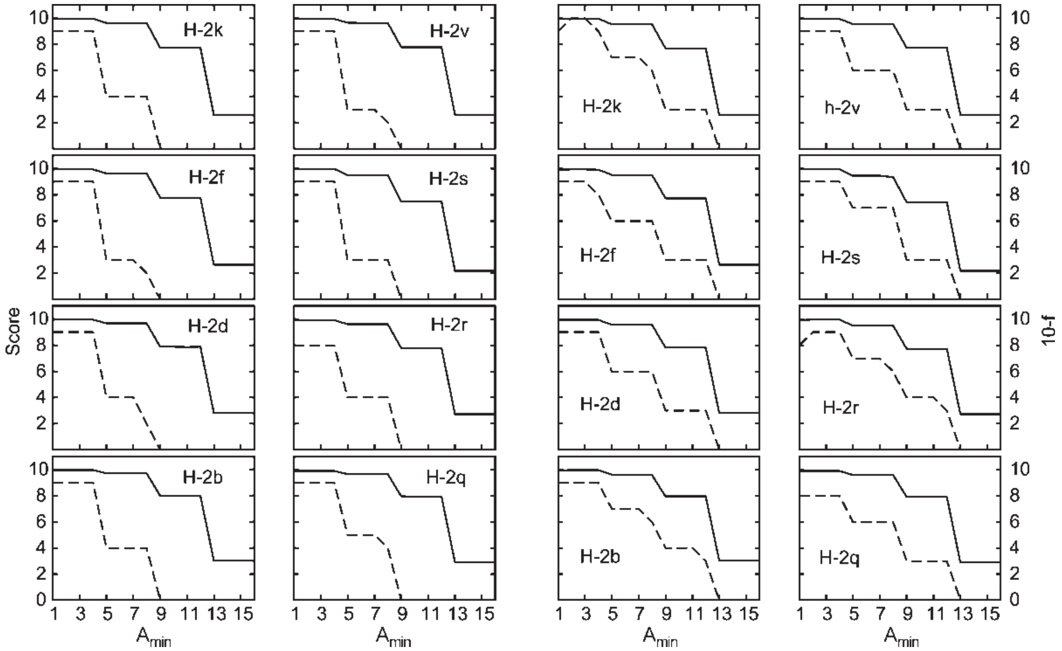

FIGURE 1 Comparison of each model's predicted repertoires to the experimental ones, for eight different haplotypes. Left 2 columns: Sequential model, Right 2 columns: Two-step selection model. The predicted repertoires had been shaped for the case of four genes, Ly49a, Ly49g2, Ly49f and Ly49i; each receptor gene varied in its binding affinity to its self-MHC-I ligand. Shown are (10−*f*), *f* being the best fit (solid lines), given instead of *f* itself in order to show the correlation between fit and score; and the best score (dashed lines), for each set of parameters. These simulations were done in the inclusive mode. The best fit and highest score were obtained with the following parameter value sets: for H-$2^b$ ($A_{min}$ = 3, $A_{max}$ = 5) and ($A_{min}$ = 4, $A_{max}$ = 7); for H-$2^d$ ($A_{min}$ = 3, $A_{max}$ = 3 or 5); for H-$2^f$ ($A_{min}$ = 3, $A_{max}$ = 5) and ($A_{min}$ = 2, $4 \leq A_{max} \leq 6$); for H-$2^k$ ($A_{min}$ = 2, $4 \leq A_{max} \leq 6$) and ($A_{min}$ = 3, $A_{max}$ = 6); for H-$2^q$ ($A_{min}$ = 2, $A_{max}$ = 3) and ($A_{min}$ = 3, $A_{max}$ = 4); for H-$2^r$ ($A_{min}$ = 2, $2 \leq A_{max} \leq 4$) and ($A_{min}$ = 3, $3 \leq A_{max} \leq 5$); for H-$2^s$ ($A_{min}$ = 1, $2 \leq A_{max} \leq 6$); and ($A_{min}$ = 2, $4 \leq A_{max} \leq 6$); for H-$2^v$ ($A_{min}$ = 2, $A_{max}$ = 3 or 4) and ($A_{min}$ = 3, $3 \leq A_{max} \leq 5$).

background. As for the affinity values of receptors Ly49G2 and Ly49F, the set of receptor binding affinities which led to the best fit to experimental frequencies were such that Ly49G2 had to be a self-MHC receptor (binds H-$2^k$ with sufficiently high affinity to induce cell maturation) under both models. On the other hand, Ly49F was predicted by the selection model to be a non-self or a low affinity receptor that can be expressed only with Ly49G2 or Ly49I, but not with the Ly49A receptor; while the sequential model predicts that, in the H-$2^k$ background, Ly49F must be a self receptor, with a low affinity, that enables the cell to mature only if it expresses another receptor. Table IV presents the predicted reactivities of all eight H-2 molecules with all four indicated Ly49 receptors, under either the two-step selection or the sequential model. The table presents the predictions in the best-fit case for each MHC background, that is, with the values of $A_{min}$ and $A_{max}$ given in Fig. 1. As the best fit has usually been obtained with more than one set of $A_{min}$ and $A_{max}$, the table describes the common predictions of all sets that gave the best fit.

It is worth noting that both models predict that Ly49G2 and Ly49I bind all MHC molecules. As for Ly49F and Ly49A, the models give different results for most MHC backgrounds. Thus, the two models differ in the Ly49 receptor reactivities to H-2 molecules which they predict, although both models can give repertoire compositions similar to the experimental repertoires, under some parameter sets. As knowledge of Ly49 receptor specificities and binding affinities grows, our predictions can be tested. These predictions were generated from the best fit cases to the data by Hanke *et al.* (2001); other data might generate different predictions. Obviously, slightly different predictions were made by slightly lower fit cases; without sufficient data for statistical analysis, we cannot determine the relative plausibility of these predictions.

### Predicted Repertoires of Transgenic Mice

It is clear from previous studies (Held *et al.*, 1996; Held and Raulet, 1997; Salcedo *et al.*, 1997; Hanke *et al.*, 2001) that MHC molecules impact Ly49 co-expression. In most cases MHC interaction results in a decreased receptor co-expression compared to that in class I-deficient mice. Interestingly, receptor co-expression is limited by interaction with MHC class I molecules, even if the particular MHC haplotypes used do not detectably bind the receptor affected by them. For example, Ly49F does not detectably bind H-$2^f$ or H-$2^r$, but co-expression of Ly49A and Ly49F was inhibited in both MHC backgrounds (Hanke *et al.*, 2001). In the same study, the expression of an Ly49A transgene in the H-$2^f$ background reduced the expression of Ly49G2, Ly49F and Ly49I, which do not bind H-$2^f$, to an extent similar to the reduction caused by the same transgene in H-$2^d$ mice where Ly49G2, Ly49F and Ly49I are self-specific receptors. Similarly, Fahlén *et al.* (2001) found that in Ly49C-transgenic mice on H-$2^b$ MHC background, transgene expression caused a decrease in the expression of Ly49G2 and Ly49D, although these

TABLE IV MHC-I reactivities to Ly49 receptors according to the selection and the sequential models*

| MHC | | Ly49A | Ly49G2 | Ly49F | Ly49I |
|---|---|---|---|---|---|
| $H\text{-}2^b$ | Experimental results | *X*? | X? | X? | √[†] |
| | Selection simulation | √[‡] | √ | √[‡] | √ |
| | Sequential simulation | *X* | √ | *X*[¶] | √ |
| $H\text{-}2^k$ | Experimental results | √ | X? | X? | √ |
| | Selection simulation | √ | √ | *X*[‖] | √ |
| | Sequential simulation | √[§] | √ | √[§] | √ |
| $H\text{-}2^q$ | Experimental results | √ | X? | X? | √[†] |
| | Selection simulation | √[**] | √ | √[#] | √ |
| | Sequential simulation | *X*[‖] | √ | *X*[‖] | √ |
| $H\text{-}2^d$ | Experimental results | √ | √[†] | √[†] | √[†] |
| | Selection simulation | √[‡] | √ | √[‡] | √ |
| | Sequential simulation | √[§] | √ | √[§] | √ |
| $H\text{-}2^f$ | Experimental results | √ | *X*? | *X*? | *X*? |
| | Selection simulation | √[††] | √ | *X*[‖] | √ |
| | Sequential simulation | *X*[‖] | √ | *X*[‖] | √ |
| $H\text{-}2^r$ | Experimental results | √ | X? | *X*? | √ |
| | Selection simulation | √ | √[‡‡] | √[‡‡] | √ |
| | Sequential simulation | √ | √[§] | √[§] | √ |
| $H\text{-}2^s$ | Experimental results | √[†] | X? | X? | √ |
| | Selection simulation | √ | √ | √ | √ |
| | Sequential simulation | √ | √ | *X*[‖] | √ |
| $H\text{-}2^v$ | Experimental results | √ | X? | X? | √[†] |
| | Selection simulation | √ | √ | √[‡] | √ |
| | Sequential simulation | √[§] | √ | *X* | √ |

√ represents a self receptor, while *X* represents a non-self receptor. *X*? refers to cases where no specificity to the particular MHC background was detected, so even if the indicated receptor binds that MHC molecule, that binding was probably under the experimental detection threshold.
* Data of experimental reactivities was taken from Hanke et al. (1999).
[†] Weak interaction.
[‡] Self receptor with affinity that enables the cell to mature only if it expresses Ly49G2 or Ly49I as well.
[¶] Non-self receptor, or a self receptor with low affinity that enables the cell to mature only if it expresses receptor Ly49G2 or Ly49I too.
§ Self receptor with a low affinity that enables the cell to mature only if it expresses another receptor.
[‖] Non-self receptor, or a self receptor with low affinity that enables the cell to mature only if it expresses another receptor.
[#] Self receptor with affinity that enables the cell to mature only if it expresses Ly49G2 as well.
** Self receptor with a high affinity that kill the cell in the selection, or self receptor with a medium affinity that enables the cell to mature only if it expresses Ly49F as well.
[††] Self receptor with affinity that enables the cell to mature only if it expresses Ly49F as well.
[‡‡] Cells that express Ly49G2 receptor with Ly49F receptor would not have sufficient affinity level in order to pass the selection. Cells that express this receptor with Ly49A or Ly49I would have too high affinity and they also would die in the selection.

receptors were assumed to have no ligand in $H\text{-}2^b$ mice. These results were taken to support the sequential model, which predicts that the transgene will inhibit expression of self MHC specific receptors as well as non-self specific receptors, while the selection model predicts that the transgene will not affect the expression of non-self receptors since there is a selection only against receptors that interact with the host's MHC.

We thus proceeded to use our simulations to compare the frequencies of cells expressing self receptors, non-self receptors and pairs of receptors in two cases for each model: non-transgenic mice, and transgenic mice in which one of the self receptor genes is expressed in all NK cells at an early stage of development, before the cell normally begins to activate different Ly49 receptor genes. The resulting repertoire, for one arbitrary set of parameters, is shown in Fig. 2. Both models predict a reduced co-expression of self-specific receptors in transgenic mice, while expression of non-self-specific receptors in transgenic mice was reduced only in simulations of the sequential model. When we examine the effect of the transgene on the co-expression of a pair of one self receptor and one non-self receptor, or the co-expression of two self-specific receptors, we see that both models predict that transgene expression would result in reduced co-expression of the two other receptors, even if one of them is non-self-specific receptor. These predictions of the two models are reasonable, since a general reduction of the frequencies of cells expressing a specific self receptor in both models would imply a reduction of the frequencies of cells expressing the indicated self-receptor with a non-self receptor, even if the general frequency of the non-self receptor does not change (as in the selection model). These results are in line with our previous predictions and do support the sequential model.

### DISCUSSION

In this study we have used computer simulations of the process of NK cell development to evaluate the two

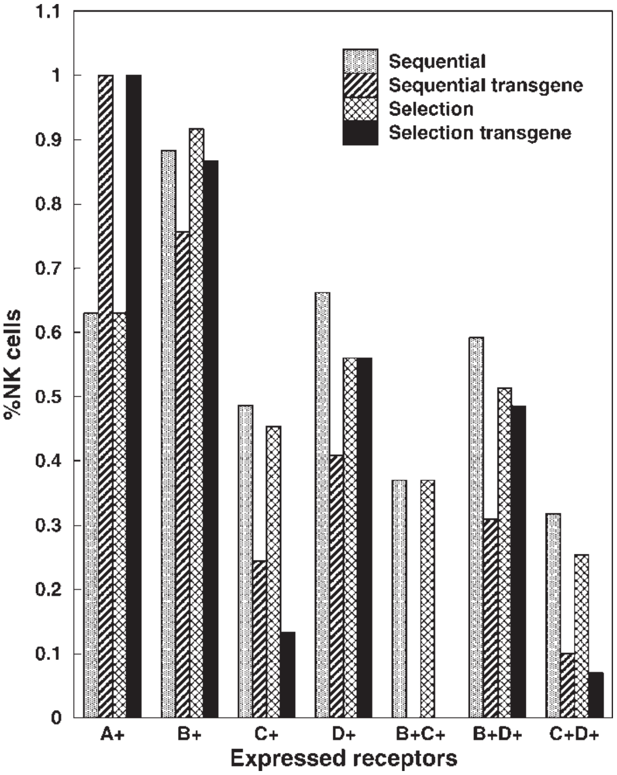

FIGURE 2 Predicted effect of a self-MHC receptor transgene, (A), on the frequencies of NK cells expressing other inhibitory receptors according to the two education models, for the case of four receptor genes: **A**- self, $p_A = 0.27$, $A_A = 2$, **B**- self, $p_B = 0.62$, $A_B = 2$, **C**- self, $p_C = 0.2$, $A_C = 1$, **D**- non-self, $p_D = 0.56$, $A_D = 0$, $A_{\min} = 3$, $A_{\max} = 4$.

competing models of NK cell "education", that is, of how developing NK cells are selected into the mature NK cell repertoire. The studies presented here make specific predictions concerning receptor expression patterns in the mature NK cell repertoire generated under each of the competing models. While available data are yet insufficient to unequivocally refute either model, our calculations predict the receptor affinity or avidity levels which would result in the observed receptor expression pattern under each model. Thus, our predictions may be used in the future to design experiments which would, in combination with our results, serve as more decisive tests for the current theories on NK cell development.

The input parameters for our models are the pre-selection receptor expression frequencies. Since these frequencies are not known, we have used the receptor expression frequencies from MHC deficient mice. However, it is known that $\beta_2 m^-$ deficient or TAP$^-$ deficient mice still exhibit some MHC-I expression, and hence regarding the NK cell repertoires in these mice as pre-selection repertoires is an approximation. In our view, this is a reasonably good approximation, as it is plausible to assume that the low levels of "leaky" MHC expression in MHC-deficient mice do not exert the same selective forces on developing NK cells as experienced by cells developing in normal mice.

Our predictions are based on the best fit to the most comprehensive data set published so far, namely, that of (Hanke *et al.*, 2001). Granted, these data are still limited, especially in the sense that only one data point (frequency measurement) was published for each receptor or pair of receptors in each MHC background. Thus, our predictions are only as good as the data on which they are based. Different data sets, with possibly more extensive expression patterns measurements (e.g. the frequencies of co-expression of more than two receptors), may have resulted in different predictions. However, the models we used are easily adaptable, and are innovative in their ability to generate testable predictions concerning NK cell education. We have focused on predicting receptor-ligand affinities, the measurements of which are less demanding than that of repertoire compositions, in an effort to increase the predictive value of our studies. Testing our predictions, and using them to evaluate the relative plausibility of the two selection models, should now be straightforward.

Data on the co-expression of more than two receptors will be very useful in trying to understand selection mechanisms. The detection of up to six different receptors expression on individual NK cells by single cell RT-PCR (Kubota *et al.*, 1999) does not necessarily imply that all the receptors are expressed on the cell's surface at levels sufficient for significant influence on the selection process. Indeed, the RT-PCR detection frequency on NK cells expressing individual Ly49 is slightly higher than that determined by flow cytometry (Held and Raulet, 1997). One reason for that could be that cell surface expression levels of Ly49 receptors are influenced by MHC class I molecules expressed on surrounding cells (Kåse *et al.*, 1998). Thus, even though a cell expresses high levels of RNA for a certain Ly49 receptor, posttranscriptional events could limit their expression at the cell surface. This has been most clearly demonstrated in MHC transgenic mice. For example, in H-2$^d$-negative mice, Ly49A receptors are expressed to high levels on a subset of NK cells. When the corresponding MHC class I ligand is introduced, NK cells expressing Ly49 A receptors to high levels downmodulate their receptors via as yet unknown mechanisms (Kåse *et al.*, 1998). Such changes in cell surface expression affect NK cell function and will therefore be important parameters to take into account in the future development of mathematical models for NK cell selection and repertoire formation.

### METHODOLOGY: MODELS AND SIMULATIONS

Vance and Raulet (1998) performed a simple calculation of the post-selection expression frequencies of various combinations of NK cell receptors, for the case in which the pre-selection expression frequencies of all receptors are equal. In our previous study (Salmon-Divon *et al.*, 2002), we have extended these calculations to the general case in which the pre-selection expression frequencies of different receptors are not necessarily equal. Here, we extend the models further, to the case in which different receptors may have different affinities of binding to their MHC ligands.

Since actual values of binding affinities of all NK cell inhibitory receptors to their MHC ligand have not yet been measured, we treat binding affinities in the following way. Receptor-ligand binding affinities are classified into one out of five possible affinity levels: 0 (no binding), 1 (low affinity), 2 (medium affinity), 3 (high affinity) or 4 (very high affinity). This is a discrete approximation for the continuum of actual affinity values. One could approximate binding avidities—avidity being defined as the product of affinity and receptor expression level—in a similar way; at this point there is no data on receptor expression levels in all cases, so that our discrete levels can represent either affinity or total binding avidity. The affinity of the receptor numbered $i$ to its ligand is thus denoted by $A_i$. As mentioned above, an NK cell can express more than one Ly49 receptor; however, the value of receptor binding affinity to MHC molecules which is sufficient to transduce inhibitory signals into the NK cell is not known. To obtain the total affinity experienced by an individual NK cell, $A_{\text{total}}$, we sum over all binding affinities $A_i$ of self-inhibitory receptors, $r_i$, that belong to the set $k_i$ expressed by the cell, i.e.

$$A_{\text{total}} = \sum_{r_i \in k_i} A_i$$

### The Two-step Selection Model with Variable Receptor Binding Affinity

To implement the two-step selection model, we define the following variables: $A_{\min}$ represents the minimum value of receptor-MHC binding affinity required for NK cell maturation, and $A_{max}$ represents the maximum allowed affinity.

We designate by $C_{pas}$ the set of all expression patterns $c_j$ in the pre-selection repertoire that cause a cell to pass the selection process i.e.

$$\{C_j | A_{min} \leq A_{total(C_j)} \leq A_{max}\}$$

Cells that are not included in this subset will be deleted and will not contribute to the final, post-selection mature NK cell repertoire.

To obtain $f(C_{pas})$, the pre-selection frequency of expression patterns of cells that successfully passed the selection process, we simply sum up the pre-selection frequencies $f(c_i)$ of all expression patterns which belong to the set $C_{pas}$:

$$f(C_{pas}) = \sum_{i=1}^{|C_{pas}|} f(c_i)$$

The two-step selection model is implemented in a computer program (Fig. 3), which first generates the table of all possible expression patterns (combinations of receptors) and calculates their pre-selection frequencies as described in Salmon-Divon *et al.* (2002). Each case in the table has its total binding affinity to self MHC class I calculated by summarizing all binding affinities of expressed self receptors. The program then applies the selection rules to the pre-selection repertoire: those cases which have an appropriate total affinity, $A_{min} \leq A_{total} \leq A_{max}$, will pass the selection. The program's output is the frequencies of cells in the post-selection repertoire that possess different patterns of surface receptor expression.

### The Sequential Model with Variable Receptor Binding Affinity

As mentioned above, the main postulate of the sequential model is that engagement of self-specific receptors by class I molecules terminates the activation of additional receptor genes. In this version of the sequential model, we presume that the interaction affinity between the receptor and MHC molecules must exceed a specific threshold, $A_{min}$, in order to terminate additional receptor gene activation, so that in some cases, signaling through several self-specific receptors is necessary. Low receptor affinity or low cell surface levels might lead to such a situation. Thus, we assume that high affinity between the receptor and the MHC ligand represents a strong interaction, leading to an appropriate signal which exceeds the signal threshold and resulting in maturation of the cell. However, low affinity will not be sufficient to prevent new receptor gene expression and, in that case, more than one self-specific receptor will be necessary for cell maturation.

Analytical prediction of the final NK cell repertoire in the case in which self receptors vary in their binding affinity to self-MHC molecules is very difficult to perform, due to the large range of possible affinity values. Therefore, we use computer simulations to predict NK cell repertoire formation in this case. The simple computer simulation of the sequential model creates a NK cell repertoire of $10^6$ cells. After a cell is created, it begins to activate Ly49 receptors, and continues to do so until total binding to self-MHC receptors exceeds the required threshold (Fig. 4). Each expressed self receptor has a specific binding affinity to self MHC class I molecules, and contributes to the total binding affinity of the cell to MHC molecules. Once a cell matures, its surface receptor expression is recorded. The output of the simulation is

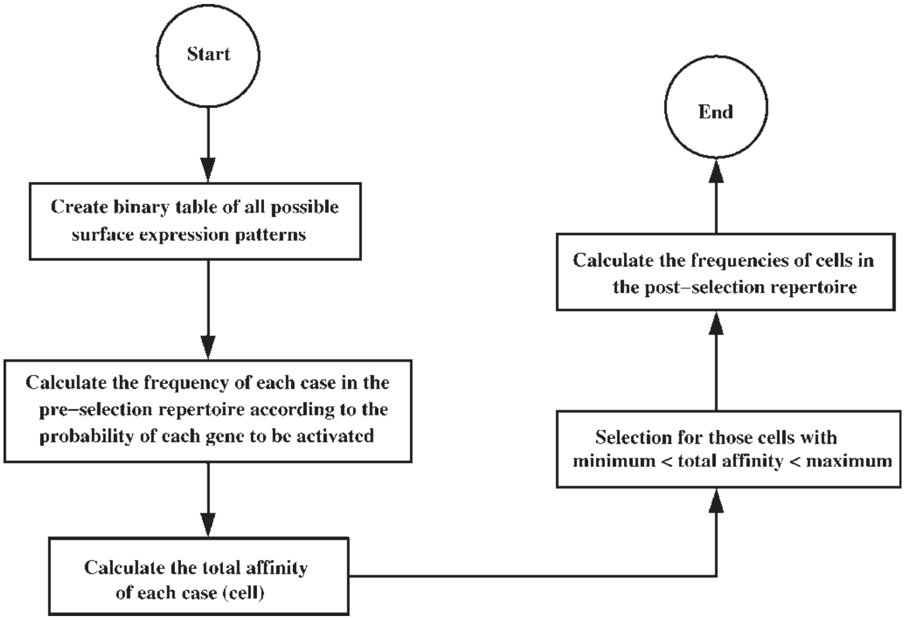

FIGURE 3 Flow chart of the program implementing the two-step selection model. (The program is available from the authors upon request.)

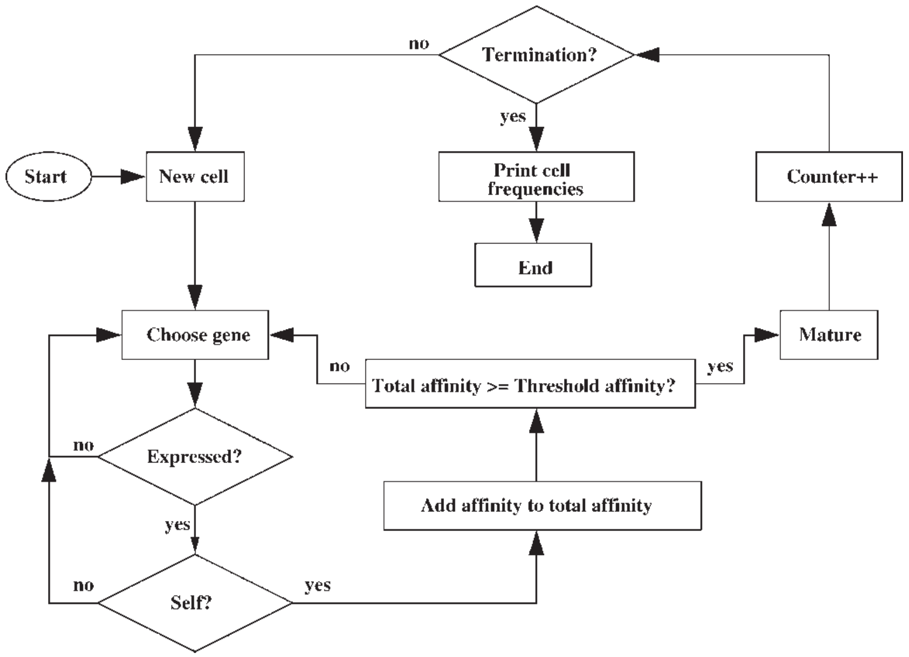

FIGURE 4 Flow chart of the sequential simulation. (The program is available from the authors upon request.)

the frequencies of cells in the post-selection NK cell repertoire that express different patterns of surface receptors.

### Fit of Simulation Results to Experimental Data

Using our simulations, we can compare the predicted repertoires that had been shaped according to each model, and obtain insight on the parameters governing NK cell repertoire. In addition, these simulations enable us to search for the sets of parameters, such as receptor binding affinities to self-MHC molecules, which lead to a final repertoire that is close to that found in laboratory experiments.

In order to decide which model gives the best fit to the data, we examined two statistical parameters: the best fit and the best score. The fit, $f$, is the sum of the squared deviations of the simulated points, $S_i$, from the experimental ones, $E_i$:

$$f = \sum_{i=1}^{n} (E_i - S_i)^2$$

The smaller the fit, the closer our results are to the experimental ones.

Note that, since the data we compared our results to in this paper included only one data point per receptor pair per MHC background, we cannot use any statistical tests. Hence we cannot say whether fits generated by different parameter sets are *significantly* different from the experimental results. An alternative cutoff between different simulations is defined by the score. The score is the number of simulated data points that deviate by no more than $\pm$ 10% from the experimental ones. That is, if a particular data set has, e.g. six points (frequencies of expression of six possible receptor combinations), then the score may vary between zero and six.

### *Acknowledgements*

The authors are grateful to Ms. Hanna Edelman for assistance with graphics. Supported in part by Israel Science Foundation—The Dorot Science Fellowship Foundation grant number 759/01-1, The Yigal Alon Fellowship, and a Bar-Ilan University internal grant (to R.M.)

### *References*

Anderson, S.K., Ortaldo, J.R. and McVicar, D.W. (2001) "The ever-expanding ly49 gene family: repertoire and signaling", *Immunol. Rev.* **181**, 79–89.

Bix, M., Liao, N.-S., Zijlstra, M., Loring, J., Jaenisch, R. and Raulet, D. (1991) "Rejection of class I MHC-deficient haemopoietic cells by irradiated MHC-matched mice", *Nature* **349**, 329–331.

Colonna, M. and Samaridis, J. (1995) "Cloning of immunoglobulin-superfamily members associated with HLA-C and HLA-B recognition by human NK cells", *Science* **268**, 405–408.

Fahlén, L., Lendahl, U. and Sentman, C.L. (2001) "MHC class I-Ly49 interaction shape the Ly49 repertoire on murine NK cells", *J. Immunol.* **166**, 6585–6592.

Hanke, T. and Raulet, D.H. (2001) "Cumulative inhibition of NK cells and T cells resulting from engagement of multiple inhibitory Ly49 receptors", *J. Immunol.* **166**, 3002–3007.

Hanke, T., Takizawa, H., McMahon, C.W., Busch, D.H., Pamer, E.G., Miller, J.D., D.Altman, J., Liu, Y., Cado, D., Lemonnier, F.A., Bjorkman, P.J. and Raulet, D.H. (1999) "Direct assessment of MHC class I binding by seven Ly49 inhibitory NK cell receptors", *Immunity* **11**, 67–77.

Hanke, T., Takizawa, H. and Raulet, D.H. (2001) "MHC-dependent shaping of the inhibitory Ly49 receptor repertoire on NK cells:

evidence for a regulated sequential model", *Eur. J. Immunol.* **31**, 3370–3379.

Held, W. and Raulet, D.H. (1997) "Ly49A transgenic mice provide evidence for a major histocompatibility complex-dependent education process in natural killer cell development", *J. Exp. Med.* **185**, 2079–2088.

Held, W., Dorfman, J.R., Wu, M.-F. and Raulet, D.H. (1996) "Major histocompatibility complex class I dependent skewing of the natural killer cell LY49 receptor repertoire", *Eur. J. Immunol.* **26**, 2286–2292.

Held, W., Kunz, B., Ioannidis, V. and Lowin-Kropf, B. (1999) "Monoallelic Ly49 NK cell receptor expression", *Semin. Immunol.* **11**, 349–355.

Höglund, P., Ljunggren, H.G., Öhlén, C., Ahrlund-Richter, L., Scangos, G., Bieberich, C., Jay, G., Klein, G. and Kärre, K. (1988) "Natural resistance against lymphoma grafts conveyed by H-2Dd transgene to C57BL mice", *J. Exp. Med.* **168**, 1469–1474.

Höglund, P., Öhlén, C., Carbone, E., Franksson, L., Ljunggren, H.G., Latour, A., Koller, B. and Kärre, K. (1991) "Recognition of $\beta_2$-microglobulin-negative ($\beta_2 m$-) T-cell blasts by natural killer cells from normal but not from $\beta_2 m$- mice: nonresponsiveness controlled by $\beta_2 m$- bone marrow in chimeric mice", *Proc. Natl Acad. Sci. USA* **88**, 10332–10336.

Höglund, P., Glas, R., Ménard, C., Kåse, A., Johansson, M.H., Franksson, L., Lemmonier, F. and Kärre, K. (1998) "$\beta$2-microglobulin-deficient NK cells show increased sensitivity to MHC class I-mediated inhibition, but self tolerance does not depend upon target cell expression of H-2$K^b$ and $D^b$ heavy chains", *Eur. J. Immunol.* **28**, 370–378.

Kåse, A., Johansson, M.H., Olsson-Alheim, M.Y., Kärre, K. and Höglund, P. (1998) "External and internal calibration of the MHC class I-specific receptor Ly49A on murine natural killer cells", *J. Immunol.* **161**, 6133–6138.

Kubota, A., Kubota, S., Lohwasser, S., Mager, D.L. and Takei, F. (1999) "Diversity of NK cell receptor repertoire in adult and neonatal mice", *J. Immunol.* **163**, 212–216.

Liado, N.-S., Bix, M., Zijlstra, M., Jaenisch, R. and Raulet, D. (1991) "MHC class I deficiency: susceptibility to natural killer (NK) cells and impaired NK activity", *Science* **253**, 199–202.

Ljunggren, H.-G. and Kärre, K. (1990) "In search of the 'missing self': MHC molecules and NK cell recognition", *Immunol. Today* **11**, 237–244.

Mason, L.H., Anderson, S.K., Yokoyama, W.M., Smith, H.R.C., Winkler-Pickett, R. and Ortaldo, J.R. (1996) "The Ly-49D receptor activates murine natural killer cells", *J. Exp. Med.* **184**, 2119–2128.

Öhlén, C., Kling, G., Höglund, P., Hansson, M., Scangos, G., Bieberich, C., Jay, G. and Kärre, K. (1989) "Prevention of allogeneic bone marrow graft rejection by H-2 transgene in donor mice", *Science* **246**, 666–668.

Salcedo, M., Diehl, A.D., Olsson-Alheim, M.Y., Sundbäck, J., Van-Kaer, L., Kärre, K. and Ljunggren, H.G. (1997) "Altered expression of Ly49 inhibitory receptors on natural killer cells from MHC class-I deficient mice", *J. Immunol.* **158**, 3174–3180.

Salmon-Divon, M., Höglund, P. and Mehr, R. (2002) "Generation of the natural killer cell repertoire: the sequential vs the two-step selection model", *Bull. Math. Biol.*, In press.

Smith, H.R.C., Karlhofer, F.M. and Yokoyama, W.M. (1994) "Ly-49 multigene family expressed by IL-2 activated NK cells", *J. Imunol.* **153**, 1068–1079.

Smith, H.R.C., Chuang, H.H., Wang, L.L., Salcedo, M., Heusel, J.W. and Yokoyama, W.M. (2000) "Nonstochastic coexpression of activation receptors on murine natural killer cells", *J. Exp. Med.* **191**, 1341–1354.

Vance, R.E. and Raulet, D.H. (1998) "Toward a quantitative analysis of the repertoire of class I MHC specific inhibitory receptors on natural killer cells", *Curr. Top. Microbiol. Immunol.* **230**, 135–160.

Yokoyama, W.M., Kehn, P.J., Cohen, D.I. and Shevach, E.M. (1990) "Chromosomal location of the Ly-49(A1,YE1/48) multigene family. Genetic association with the NK1.1 antigen", *J. Immunol.* **145**, 2353–2358.

